# *N-*Butyl-2-Cyanoacrylate versus Microspheres on Weight Change and Ghrelin Expressions in Swine Bariatric Embolization Model

**DOI:** 10.1186/s42155-025-00556-9

**Published:** 2025-07-02

**Authors:** Won Seok Choi, Young Suk Park, Kun Yung Kim, Chong-ho Lee, Minuk Kim, Chang Jin Yoon, Jae Hwan Lee

**Affiliations:** 1 Present Address: Seoul National University Bundang Hospital, Seongnam, South Korea; 2 Seoul Metropolitan Boramae Hospital, Seoul National University Seoul Metropolitan Government Boramae Medical Center, Seoul, South Korea; 3 Present Address: Department of Radiology, Seoul National University College of Medicine, Seoul, South Korea

**Keywords:** Obesity, Embolization, Endovascular procedures, Stomach, Ghrelin

## Abstract

**Background:**

Obesity is a global health challenge, leading researchers to explore innovative treatments. Bariatric arterial embolization (BAE), which blocks blood flow to parts of the stomach, shows promise for weight management by affecting hunger hormones like ghrelin. This study aimed to compare the efficacy and safety of *n*-butyl-2-cyanoacrylate (NBCA) and microspheres in suppressing weight gain and ghrelin expression after BAE in a swine model.

**Materials and Methods:**

Fifteen healthy juvenile male farm pigs were randomly allocated into three groups: NBCA embolization (*n* = 5), microsphere embolization (*n* = 5), and a control group (n = 5). Embolization targeted the right, left, and short gastric arteries. Weight and fasting plasma ghrelin levels were monitored weekly for 16 weeks. Gastric endoscopy was performed 1 and 4 weeks post-BAE, and each animal's ghrelin-expressing cells in the stomach's fundus, body, and antrum were analyzed.

**Results:**

By week 16, the NBCA group showed lower weight gain (58.4 ± 17.8%) compared to that in the microsphere (114.0 ± 0.0%; *P* < .001) and control groups (123.9 ± 18.1%; *P* < .001). The NBCA group had lower mean ghrelin-expressing cell densities in the gastric fundus (*P* < .001), body (*P* = .002), and antrum (*P* = 0.003) compared to those in the control group, and lower ghrelin-expressing cell densities in the fundus compared to those in the microsphere group (*P* < .001). Endoscopy at 1-week post-BAE revealed gastric ulcers in 2 pigs in the NBCA group (40%) and all pigs (100%) in the microsphere group, which healed by week 4; no ulcers were found in the control group.

**Conclusions:**

In a swine model of bariatric arterial embolization, NBCA was more effective than microspheres in reducing weight gain and ghrelin expression in the stomach fundus, indicating its potential for managing obesity through BAE.

## Introduction

Obesity is a global health crisis tied to increased morbidity and mortality, higher healthcare costs, and lower quality of life [1, 2]. Non-surgical treatments, like lifestyle modification, pharmacotherapy, and endoscopic procedures, face limitations that hinder their efficacy and patient acceptance. These include underutilization, high costs, and complications, driving the need for alternative treatments.

Bariatric arterial embolization (BAE) has become a promising minimally invasive technique for weight reduction. It targets hunger-related hormonal pathways, particularly ghrelin production in the gastric fundus [3–7]. Weiss et al. reported a mean weight loss of 11.5% in patients with morbid obesity after BAE in a prospective trial [7]. Preclinical swine models have further elucidated how BAE suppresses ghrelin to reduce weight gain [6, 8–10].

However, there is no consensus on the optimal embolization techniques and materials, with studies showing varied weight reduction rates ranging from 6.0% to 17.2% [7, 11–15]. A recent study demonstrated that smaller microspheres (100–300 µm) had better results in suppressing weight gain and fundal ghrelin expression than larger ones (300–500 µm) [8]. Yet, smaller particles were associated with an increased incidence of gastric mucosal necrosis [8, 16].

*N-*butyl-2-cyanoacrylate (NBCA) is a well-established liquid embolic agent used in transcatheter arterial embolization, offering several advantages [17–20]. NBCA’s fluidity allows it to infiltrate target arterioles, and polymerization ensures a permanent embolization. When combined with iodized oil, it enhances radio-opacity and enables polymerization time during injection. This lets interventional radiologists precisely monitor and control the embolization. NBCA is also durable and has a low recanalization rate, making it a robust choice for embolization [18–21].

Despite these benefits, NBCA’s role in BAE remains understudied. This study aims to test whether NBCA is more effective and safer than microspheres in suppressing weight gain and ghrelin expression following selective BAE in a swine model.

## Materials and Methods

This study was approved by the institutional animal care and use committee (No: 19–0184-S1 A1) and conducted in compliance with the ARRIVE guidelines for animal research to ensure the ethical and accurate reporting of in vivo experiments [22].

Sample size estimation was performed using G*Power software (version 3.1.9.7; Heinrich-Heine-Universität Düsseldorf, Düsseldorf, Germany). Based on previous reports (8, 9), the effect size was defined as a 20% difference in weight gain between experimental and control groups. To detect this difference with a significance level (α) of 0.05 and a statistical power (1-β) of 0.80, assuming a standard deviation of 15%. The required sample size was determined to be five animals per group.

### Procedures

From February 2020 to November 2023, fifteen healthy juvenile male farm pigs (mean weight, 33.4 kg ± 3.1 kg) were enrolled in this study. Each pig was assigned a unique identification number and randomly sorted using Excel's random number generation function (Microsoft Corporation, Redmond, WA, USA). The pigs were allocated into three groups: embolization with NBCA (*n* = 5), embolization with microspheres (100–300 µm) (*n* = 5), and a control group undergoing saline infusion (n = 5). Omeprazole (40 mg) was administered to all pigs daily from 2 days before to 30 days after the BAE.

**Fig. 1:**
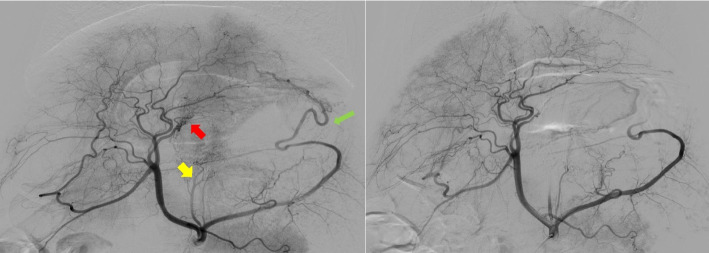
Representative digital subtraction angiograms of bariatric arterial embolization of a swine. (**a**) The pre-embolization angiography of the stomach reveals the right gastric artery (red arrow), left gastric artery (yellow arrow) and left gastroepiploic artery (green arrow) that provide blood flow to the gastric fundus for embolization. (**b**) Following the targeted embolization of the right stomach artery and the left gastroepiploic artery, the post-embolization angiography reveals the cessation of blood flow

The pigs were fasted and sedated with an intramuscular injection of tiletamine–zolazepam (5 mg/kg) and xylazine (2 mg/kg) before the BAE or sham procedure. All pigs were intubated and mechanically ventilated, with anesthesia maintained using isoflurane. Two interventional radiologists performed all the BAE and sham procedures. Percutaneous ultrasound-guided femoral arterial access was obtained, and 5-F angiographic guide catheter (Davis, Cook Medical, Bloomington, USA) under fluoroscopic guidance (Axiom Artis Zee, Simens, Forchheim, Germany) was used to perform celiac angiography with iodinated contrast media (Omnipaque, GE Healthcare, Princeton, USA) at a 4 mL/s injection rate for 5 s (Fig. 1).

After selecting the right, left, and short gastric arteries using a 1.7-F microcatheter (Progreat Lambda, Terumo, Tokyo, Japan), arteriography of each artery was performed.

In the NBCA group, the three gastric arteries were embolized with a mixture of NBCA and iodized oil (Lipiodol, Laboratoire Andre Guerbet, Aulnay-sous-Bois, France) at a volume ratio of 1:3. In the microsphere group, gastric arteries were embolized with 100–300 µm calibrated microspheres (Embosphere®, Merit Medical, South Jordan, USA), diluted by half with contrast media. The microspheres were infused until blood flow in the target artery ceased for five consecutive heartbeats. The success of the embolization was confirmed by post-embolization angiography. In the control group, saline infusion was performed.

### Follow-up examination

The animals were followed for 16 weeks to observe the long-term effects of the intervention on weight gain and Ghrelin expression. This timeframe was chosen because the subjects were healthy juvenile animals rather than obese models, and significant changes in weight gain tend to manifest more than two months post-treatment [8, 9].

The weight of the pigs was measured before BAE and weekly thereafter. Fasting blood samples were collected from the jugular vein to assess plasma ghrelin levels. Plasma ghrelin concentrations were measured using an enzyme-linked immunosorbent assay kit (Pig GHRL/Ghrelin Preproprotein, LSbio, Seattle, USA).

Gastroscopy was performed at 1 and 4 weeks after embolization by a gastroenterologist using a pediatric gastroscopy (Pentax, Denver, Colorado, USA) to evaluate the effects of embolization. Gastric ulcers with visible vessels, active bleeding, or perforation were graded as severe, while those without these features were graded as mild.

Follow-up angiography was conducted 1 day, 4 weeks, and 16 weeks after the BAE or sham procedure to evaluate the occurrence of recanalization of the gastric arteries.

### Pathology and immunohistochemistry

After euthanization, the stomachs were removed and fixed in formalin. Representative regions from the gastric fundus, body, and antrum were sampled from each animal and processed into formalin-fixed paraffin-embedded tissue blocks. Immunohistochemistry for ghrelin was performed using a rabbit polyclonal ghrelin antibody (Phoenix Pharmaceuticals, Burlingame, CA, USA) on the Benchmark XT autostainer (Ventana Medical Systems, Tucson, USA). The immunohistochemistry slides were scanned using the Aperio ScanScope (Aperio Technologies, Vista, USA), and three different high-power fields with the most active signals from the whole slide were analyzed. In these areas, the number of ghrelin-positive epithelial cells (GECs) showing cytoplasmic expression was counted, and the cell counts were presented as the mean number per high-power field.

### Statistical analysis

Statistical analyses were performed using GraphPad Prism version 5 (GraphPad Software, San Diego, CA, USA). Differences in weight changes and plasma ghrelin levels over time among groups were assessed using repeated-measures ANOVA, with normality and homogeneity of variance confirmed by the Shapiro–Wilk and Levene's tests, respectively. Post-hoc comparisons were made using the Bonferroni correction.

GEC densities were compared using one-way ANOVA with Tukey’s post-hoc test, following verification of assumptions. Mucosal ulceration and the presence of embolic materials were evaluated qualitatively and reported descriptively. A p-value of less than 0.05 was considered statistically significant.

## Results

### Procedural result

Selective embolization was successfully performed in all animals. All pigs survived until 16 weeks post-BAE without any significant procedure-related adverse events.

### Weight change

**Fig. 2:**
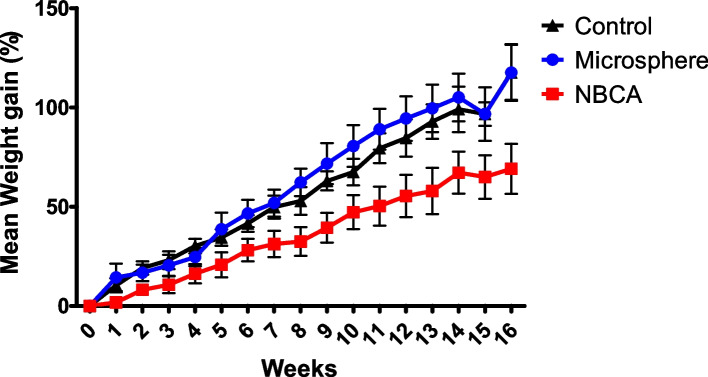
The graph displays the average percentage weight gains following bariatric arterial embolization (BAE) utilizing NBCA and 100- to 300-µm microspheres, as well as the control group that underwent sham procedures. The mean weight gain in pigs treated with NBCA was significantly lower than that of those treated with microspheres or the control group

The percentage changes in weight gain were 58.3 ± 18.1% for the NBCA group, 116.7 ± 31.9% for the microsphere group, and 125.5 ± 31.3% for the control group. The weight gain over time in the NBCA group was significantly lower than that in the microsphere group (*P* < 0.001) and the control group (*P* < 0.001). In contrast, we found no evidence of a difference in weight gain over time between the microsphere group and the control group (P = 0.35) (Fig. 2).

### Plasma ghrelin value

**Fig. 3:**
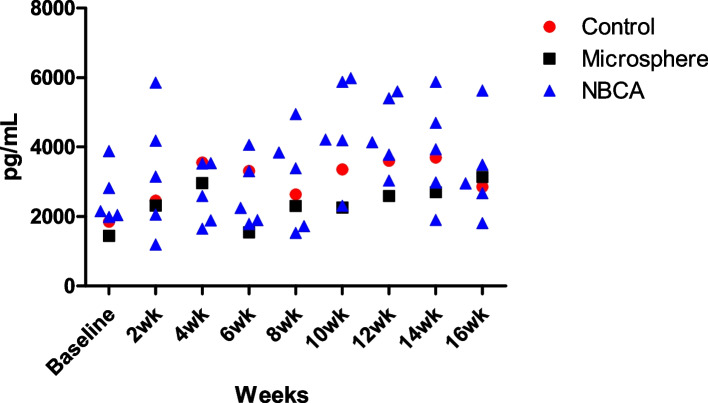
The graph shows biweekly plasma ghrelin concentrations in NBCA, microsphere, and control groups. We found no evidence of a difference in Mean plasma ghrelin levels among groups at all time points

Due to an initial optimization error in the measurement settings of plasma ghrelin concentration, data for eight samples were missing. As a result, plasma ghrelin level measurements were available for only seven pigs (NBCA, *n* = 5; microsphere, *n* = 1; control, *n* = 1). Baseline plasma ghrelin levels showed no difference over time between the NBCA, microsphere, and control groups. Weekly mean plasma ghrelin levels also showed no significant differences among the three groups at any time point (Fig. 3).

### Follow-up angiography

At the 1-day and 4-week follow-up angiographies, the right, left, and short gastric arteries showed persistent obstruction in both the NBCA and microsphere groups. However, one of the five pigs in the microsphere group showed recanalization of the right and left gastric arteries at the 16-week follow-up angiography. In contrast, all pigs in the NBCA group demonstrated obstruction of the right, left, and short gastric arteries at the 16-week follow-up angiography.

### Gastroscopic findings

**Fig. 4:**
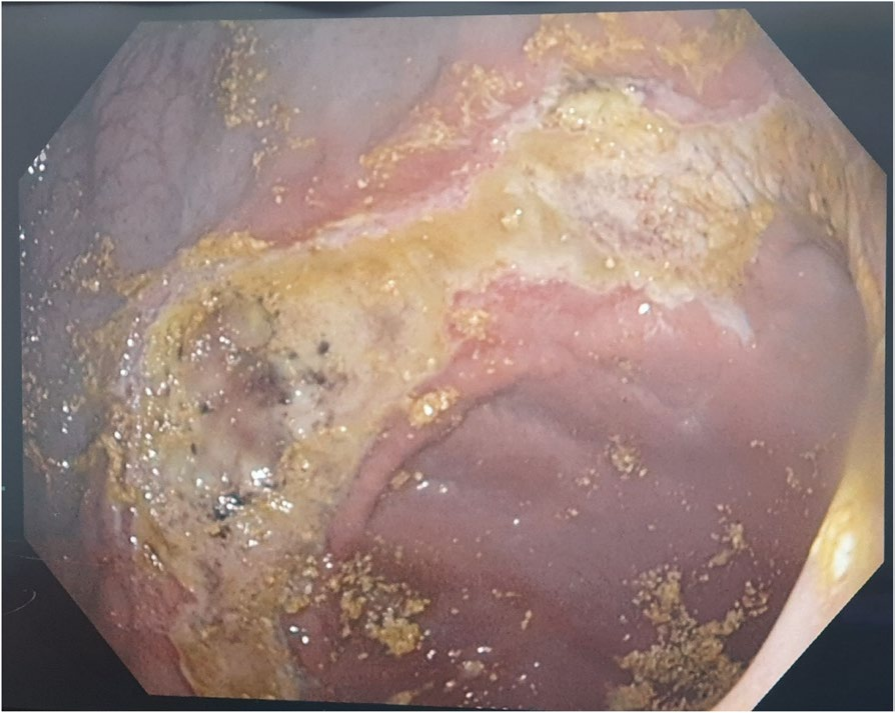
Endoscopic image of the pig stomachs of the NBCA group taken one week after embolization. There is a superficial ulcer in the high body of the stomach, near the fundus. All ulcers were mild and had completely healed by the 16 th week

At the one-week follow-up, two pigs in the NBCA group (40%) and all pigs (100%) in the microsphere group developed superficial mucosal ulceration in the gastric fundus (Fig. 4).

These gastric ulcerations were primarily located in the fundus and along the lesser or greater curvature near the fundus. No mucosal injuries were observed in the control group. A qualitative assessment graded all ulcers as mild, with no evidence of bleeding or perforation. No ulceration was detected in other gastric regions. By the 4-week follow-up endoscopy, all ulcers had completely healed, with no residual ulcers or structural distortions present.

### Histopathologic analysis

Microscopic evaluation of the stomach specimens in the microsphere group revealed the presence of microspheres predominantly localized within the submucosa and muscularis propria of the gastric body region in all subjects. Relatively lower concentrations of microsphere were observed in the fundus and antrum. In contrast, no embolic material was identified in the analyzed tissues of pigs treated with NBCA.

### Ghrelin cell density

The densities of GECs in the gastric fundus (*P* < 0.001), body (*P* = 0.002), and antrum (*P* = 0.003) were lower in the NBCA group than in the control group across all gastric regions. Similarly, the microsphere group showed lower GEC densities than those in the control group in the gastric fundus (*P* < 0.001), body (*P* = 0.003), and antrum (*P* = 0.004).

**Fig. 5:**
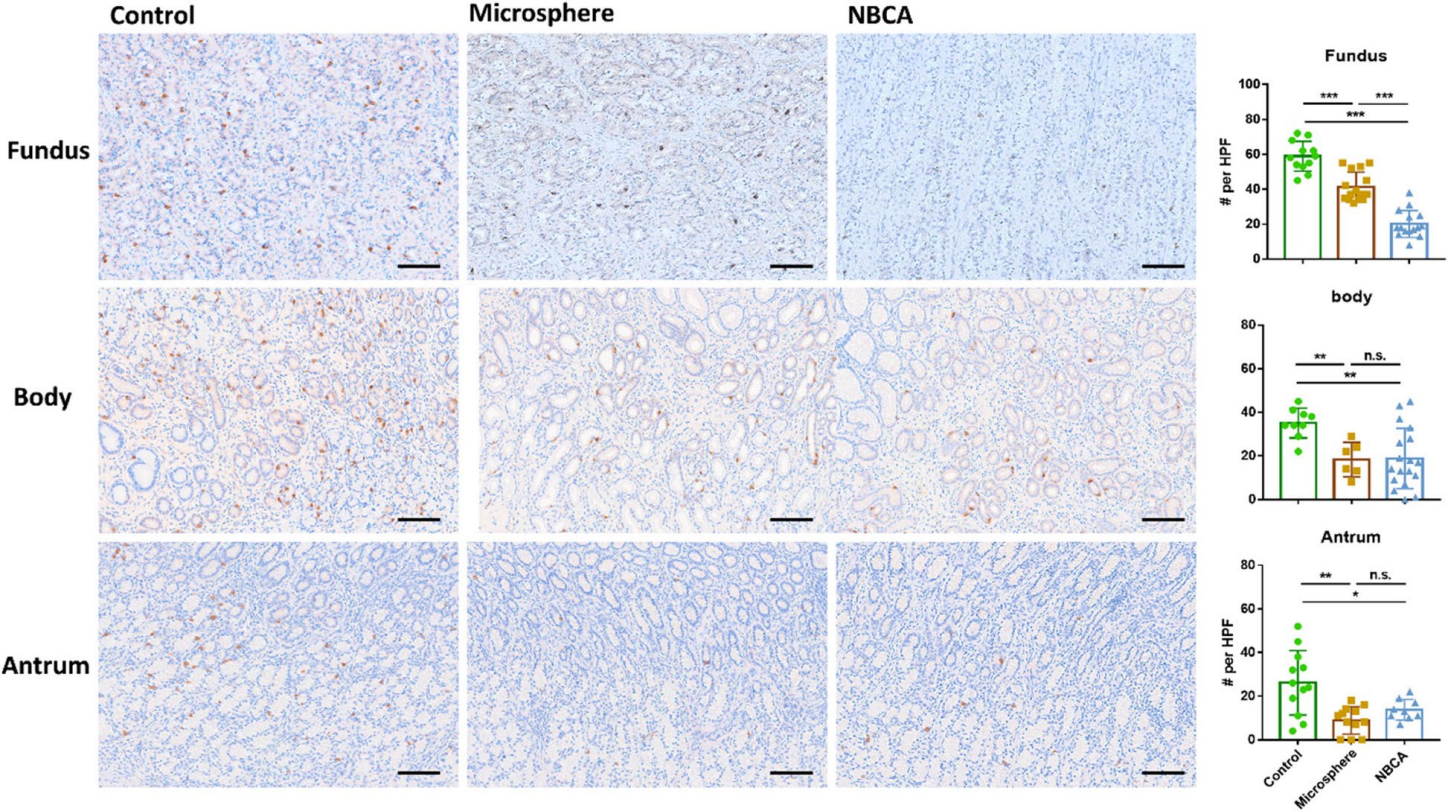
(**a**) Representative images of immunohistochemical staining for ghrelin in the gastric fundus, body, and antrum of each group (NBCA, microsphere, and control). (**b**) Bar graph depicting each group's mean count of ghrelin-expressing cells in the gastric fundus, body, and antrum. The NBCA group shows significantly lower GEC densities in the gastric fundus (*P* <.001), body (*P* =.002), and antrum (*P* =.003) compared to the control group. Additionally, the NBCA group has lower GEC densities in the gastric fundus compared to the microsphere group (*P* <.001). Data are presented as mean ± standard deviation

In the gastric fundus, the NBCA group exhibited lower densities of GECs compared to those in the microsphere group (*P* < 0.001). However, no differences in GEC densities were observed between the NBCA and microsphere groups in the gastric body (*P* = 0.830) and gastric antrum (*P* = 0.132) (Fig. 5).

## Discussion

This study demonstrated that *n*-butyl-2-cyanoacrylate is a superior embolic agent in suppressing weight gain and gastric ghrelin expression compared to calibrated microspheres. These findings offer insights into the efficacy and safety of *n*-butyl-2-cyanoacrylate as a promising embolic material for BAE.

The superior reduction in weight gain in the NBCA group compared to the microsphere group (*P* < 0.001) highlights the effectiveness of NBCA in BAE. The intrinsic disadvantage of the particles is their aggregation and clumping [9, 20, 23]. Fu et al. suggested the high friction coefficient of non-spherical polyvinyl alcohol particles (PVA) can cause aggregation, leading to proximal embolization [8]. Although calibrated microspheres offer more controlled arterial occlusion and less clumping than PVAs [24, 25], they are not visible on fluoroscopy, which may lead to non-target embolization [5].

Microspheres and NBCA differ fundamentally as embolic agents. Microspheres (100–300 µm) used in this study aggregate in vessels larger than 100 µm, reducing blood flow and providing a site for platelet/fibrin adhesion, functioning as an indirect embolic agent [26]. In contrast, NBCA directly occludes vessels through polymerization with blood and adheres firmly to the vascular endothelium. This process generates heat-promoting endothelial damage with thrombosis and rapid occlusion of the target vessel [18, 27–29].

Recanalization of embolic material is also a major challenge in BAE using microspheres. In our study, one animal in the microsphere group showed recanalization of right and left gastric arteries at the 16-week follow-up. Stampfl et al. demonstrated the recanalization of microspheres in a porcine kidney model, observing this phenomenon across all particle types and sizes, indicating that incomplete and less geometric vessel occlusion may lead to early recanalization [30]. In contrast, Woo et al. showed the NBCA group had a 2.8% vascular recanalization rate, compared to 21.5% in the PVA group [18]. The robust polymerization reaction of NBCA upon contact with blood leads to durable occlusion, enhancing its efficacy in preventing recanalization and demonstrating a superior therapeutic impact on weight gain suppression compared to the microsphere group.

Unlike the microsphere group, where all subjects developed gastric ulcers, only two pigs in the NBCA group exhibited gastric ulcers in our study. This difference can be attributed to the penetration characteristics and behavior of the embolic materials. Microspheres penetrated deeply into the submucosa, leading to ulcer formation. Additionally, when microspheres aggregate, they can generate small thromboemboli, which may cause unwanted distal embolization and ischemia. In contrast, NBCA with an appropriate concentration can occlude the targeted vessel durably at a relatively proximal site without causing distal embolization. This targeted occlusion might reduce perfusion to the target lesion while preserving microcirculation, thereby potentially minimizing the risk of ulceration (32).

The relatively lower density of GECs in the fundus of the NBCA group also supports this hypothesis, correlating with the effective suppression of weight gain. Unlike previous reports, the microspheres did not inhibit weight gain despite exhibiting lower GEC density than the control group [8]. NBCA may effectively reduce fundal perfusion where GECs were abundant, resulting in meaningful inhibition of weight gain. Consistent with recent studies [8, 9], serum ghrelin levels did not differ significantly among the groups in this study. This may be due to ghrelin's naturally dynamic secretion pattern and difficulty distinguishing between acylated and des-acyl forms of ghrelin [8, 9, 31].

This study has limitations. First, using healthy young swine as the animal model may not accurately represent the complex physiological and pathological conditions in human patients with obesity, thus potentially limiting the generalizability of the results. Second, the evaluation of the degree of embolization by NBCA was limited. Histopathologically, detecting or estimating NBCA in tissue after embolization is complex. Further studies of the effect of NBCA on tissue during embolization are warranted. Third, suppressing weight gain and weight loss are not physiologically equivalent processes, especially regarding durability. A clinical trial that thoroughly controlled for confounding factors would provide a more comprehensive understanding of the effects of NBCA in BAE. Fourth, there was a partial loss of plasma ghrelin data due to an initial measurement optimization error, resulting in missing data for eight samples. However, the analysis of the available data was coherent with previous studies [8, 19], supporting the validity of our findings.

In conclusion, our study demonstrated that the *n*-butyl-2-cyanoacrylate group achieved greater reductions in both weight gain and ghrelin expression in the stomach than those in the microsphere group in a swine model of bariatric arterial embolization. These findings support further exploration and potential clinical application of *n*-butyl cyanoacrylate in obesity treatment, focusing on optimizing procedural techniques to minimize gastrointestinal side effects.

## Data Availability

The data generated and analyzed during this study are available from the corresponding author upon reasonable request.
